# Assessing the Growth of Ethical Banking: Some Evidence from Spanish Customers

**DOI:** 10.3389/fpsyg.2017.00782

**Published:** 2017-05-24

**Authors:** Fernando E. Callejas-Albiñana, Isabel Martínez-Rodríguez, Ana I. Callejas-Albiñana, Irene M. de Vidales-Carrasco

**Affiliations:** ^1^Department of Spanish and International Economy, Econometrics, History, and Economic Institutions, University of Castilla-La ManchaCiudad Real, Spain; ^2^Department of Psychology, University of Castilla-La ManchaCiudad Real, Spain; ^3^Department of Political Economy and Public Finance, Economic Statistics, Business, and Economic Policy, University of Castilla-La ManchaCiudad Real, Spain

**Keywords:** ethical banking, traditional banking, banking consumers, deposits and loans

## Abstract

Aristotle, who, having predated Adam Smith by 2000 years, deserves to be recognized as the world’s first economist (Solomon, 1995), distinguished between two different senses of what we call *economics*: *oikonomikos*, or household trading, which he approved of and considered essential to the working of any even slightly complex society, and *chrematisike*, or trade for profit, which he considered selfish and utterly devoid of virtue, calling those who engaged in such practices “parasites”. Of course, consumers do not purchase and invest for solely economic reasons (Polanyi, 1944). Interest in ethics in economics has been the subject of continuous study. In this regard, the recent financial crisis has had not only economic, but also social, psychological, political, and ethical consequences, which have impacted the financial and banking system. Consumers are no longer drawn only by the economic return but also by ethical factors. Ethical banking is on the rise. This paper aims to explain the reasons for the growth in ethical banking and to answer the following questions: can banking consumers-investors change the characterization of the banking system? Can ethical banking gain ground on traditional banking? And is ethical banking really effective? To this end, it will examine the Spanish case, using econometric causal regression models to identify the reasons why consumers decide to invest in ethical banking and determine its role in the Spanish economy.

## Introduction

Ethics in finance has long been a subject of study, and today it is being given increasing attention. It seeks to incorporate moral, transparent, fair, and sustainable considerations into the decisions of an ever-growing number of banking consumers. The Goldman Rule that defends the pursuit of profitable opportunities, regardless of the effects on others (Watkins, 2011) appears to be disappearing in general consumption and (albeit to a lesser degree) in the consumption of financial products too.

Formally, ethical banking first emerged in its present-day role as a trader with the founding of the first ethical bank, Triodos Bank, in 1971, following a 1968 study group meeting in Holland. They sought a way to promote a more rational and conscientious way of using money and, finally in 1971, they set up the Stichting Triodos Foundation (Triodos Bank, 2008–2015:149).

On the supply side these banks take ethical responsibility into account when determining which products – financial or otherwise – to offer consumers, thereby supplying the market with a responsible offer based on trust in those who receive and manage consumers’ savings (Green, 1989). This means companies are ethically liable and are not protected by limited liability from the consequences of their actions. The role of bankers is to administer funds based on the trust of those who ask them to administer their money, and they should thus loan this money responsibly.

From this perspective, the severe financial crisis, which began in the summer of 2007 in the U.S. real estate sector and continues, even today, to affect the real economy of many developed countries, had not only economic causes, but also psychological, social, political, and ethical ones (Fernández, 1994:402). The latter include a number of ethical failures (Calvo and Mingorance, 2012) that both led to the crisis and helped to make it as deep, severe, and enduring as it has been. These failures can be divided into three distinct groups (Argandoña, 2012:2). First, there were *individual moral failures*, as witnessed by the widespread inappropriate, sometimes even criminal, behavior so often on display in the pre-crisis environment of high economic growth, abundant liquidity, low interest rates, and extraordinary opportunities to profit: concealment of information, false advertising, the proliferation of unnecessary transactions to generate higher commissions, inadequate risk assessments, high indebtedness, etc. Second, there were *ethical failures related to management or governance*, such as the many cases of poor governance and lack of professional competence by directors, senior executives, and analysts at organizations ranging from commercial and investment banks to hedge funds (Ropero and Hurtado, 2015), monolines, rating agencies, supervisory bodies, central banks, and governments. Finally, third, there were *social ethics failures*, since social conditions were created that encouraged, or at least failed to stop, inappropriate behavior at the personal and organizational levels that notably interfered with the proper functioning of the legal, institutional, and social corrective mechanisms that, in other conditions, would have prevented the moral consequences of such ethically reprehensible behavior.

Clearly, the recent systemic crisis has jeopardized global financial stability by disrupting the efficient allocation of savings and investment – the drivers of economic growth, job creation, progress, and social welfare – and, ultimately, threatening continued public trust in the financial system. Indeed, in recent years, financial systems, including Spain’s, have largely distanced themselves from their traditional role of meeting the real economy’s needs. The severe financial crisis generated a high degree of uncertainty and volatility in the international markets due, first, to the liquidity and solvency problems that many institutions were experiencing. The situation was subsequently compounded by deep economic recessions and sovereign debt crises resulting from the significant debt governments incurred as a consequence of the implementation of fiscal stimuli and measures to support distressed banks in an environment of high external leverage of the private sector (Argandoña, 2012).

In short, the economic and financial crisis has underscored the need to embrace an investment philosophy based on greater transparency, a greater presence of ethical values, and better and broader risk management. Numerous intellectuals have called for the necessary proliferation of ethical and moral principles in the economic and financial world. However, these principles do not arise spontaneously, but rather in response to the problems generated by the magnitude and frequency of business scandals and the various economic and financial crises we have witnessed in recent years, for which there has never been a politically and socially acceptable solution from a neoliberal perspective (Calvo, 2012:7). The development of an ethical culture in the financial world has resulted in the establishment of new national and international regulatory standards that help to strengthen the regulation, supervision, and risk management of the global financial system, while at the same time eliminating or reducing the harmful effects of ethical conflicts on society as a whole.

## Materials and Methods

### Conceptual Framework

The implementation of new national regulatory standards in the financial system, with greater international coordination, has primarily sought to achieve (Aspachs-Bracons et al., 2010:5): greater and better capitalization of banks, more stringent control of liquidity and solvency levels, increased transparency in financial agreements and securitizations, and greater control of risk and the level of leverage assumed by the industry. At the same time, it has revealed the need to establish a new multilateral pillar in order to strengthen the regulation, supervision, and risk management of the international financial system, thereby preventing new systemic crises and mitigating their global impact should they occur.

#### The Current Traditional Banking System

In response to the aforementioned guidelines, the Basel Committee on Banking Supervision developed a comprehensive set of reforms known as Basel III with a view to improving the banking industry’s ability to absorb shocks caused by economic or financial stress, whatever the source, improving banks’ risk management and governance, and strengthening banks’ transparency and disclosures (Rodríguez de Codes Elorriaga, 2010; Bank of International Settlements, 2016). As the new capital and liquidity measures significantly tighten bank regulation, a broad transitional period was established, to last from January 1, 2013, to January 1, 2019, so that the changes could be implemented gradually. These measures were part of a broader reform process, arising from the action plan that the G20 approved at the Washington Summit held on November 14 and 15, 2008, to provide global solutions to the crisis and improve international cooperation.

Subsequently, at the G20’s London Summit, held on April 2, 2009 (El País, 2009), in addition to a broad package of measures aimed at restoring growth and employment, strengthening financial institutions, and promoting global trade and investment, the group agreed to establish a new Financial Stability Board (FSB). The FSB would succeed the Financial Stability Forum (FSF) and would consist of the G20 countries, Spain, and the European Commission. Its actions, which it performs in close collaboration with the IMF and the Basel Committee, are mainly aimed at maintaining global financial stability and the openness and transparency of the financial sector. To this end, its members commit to implementing international financial standards and undergoing periodic reviews of their compliance with these obligations (Banco de España, 2016).

Meanwhile, at the European Union level, two new institutions were created (Field and Moreno García, 2010:114). The first was the European Systemic Risk Board (ESRB), an independent body responsible for the macroprudential supervision of the Union’s financial system, which aims to identify and prioritize systemic risks and, where applicable, issue recommendations for action and monitor their implementation. The second was the European System of Financial Supervision (ESFS), tasked with microprudential supervision, i.e., the supervision of individual institutions. The ESFS is made up of the national financial supervisors and three new European supervision authorities: the European Banking Authority (EBA), the European Securities and Markets Authority (ESMA), and the European Insurance and Occupational Pensions Authority (EIOPA). These European authorities were set up based on existing European committees in order to harmonize standards, promote the application of European legislation, and, when necessary, help settle disputes between national supervisors.

Finally, in the summer of 2012, the leaders of the European Union undertook to advance on the creation of the European Banking Union (EBU). The EBU first emerged as a step toward financial integration, i.e., toward a single market for financial services and, ultimately, toward the perfection of the construction of the euro by re-establishing a properly functioning monetary policy in the eurozone and restoring confidence in the European banking sector (Abascal Rojo et al., 2014). To achieve these goals of greater and better use of financing sources and higher levels of competition, efficiency, technology, and diversification, the EBU is organized around three main pillars, to which a fourth might be added:

(1) A single supervisory mechanism (SSM), defined as a European financial supervision system made up of the European Central Bank (ECB) and the national supervisors of the eurozone states and of those other countries that choose to join it. Since November 4, 2014, when it assumed its duties, it has been responsible for ensuring the safety and soundness of the European banking system and for increasing financial integration and stability in Europe.
(2) A single resolution mechanism (SRM), approved by the European Parliament on May 15, 2014, whose primary aim is to ensure the efficient resolution of banks facing serious difficulties with minimal costs to taxpayers and the real economy.
(3) A European deposit insurance scheme (EDIS), which, however, remains far from becoming a reality today, despite the approval of a Directive on deposit guarantee schemes (Directive 2014/49/EU of the European Parliament and of the Council of 16 April 2014 on deposit guarantee schemes).
(4) A single regulatory system, which, in order to achieve the ultimate goal of a more resilient, transparent, and efficient European banking sector, pursues the more specific goals of providing harmonized prudential rules for EU institutions, unifying the regulatory framework of the EU financial system to complete the single market in financial services, and ensuring uniform application of Basel III.

However, this financial regulation, at both the national and supranational level, has generally proved insufficient to prevent the numerous ethical conflicts that arise in the world of finance. This is mainly due to the great complexity of financial activities, which facilitates avoidance of the undertaken obligations. Consequently, the various players in these markets must also engage in self-regulation. This can be pursued, first, within the financial institutions themselves, through the establishment of codes of ethics or of conduct, which have enabled significant improvements in institutional transparency, and through a commitment to corporate social responsibility, with the aim of managing the impact these institutions’ activities have on their customers, employees, shareholders, local communities, the environment, and society at large. Second, it can be achieved through appropriate financial training and education that equips the recipients of the training with the knowledge, skills, behavior, values, and aptitudes they need to make sensible and informed financial decisions, in addition to leaving them better positioned to deal with the basic financial challenges they will encounter over their lives. Indeed, to paraphrase Melé, “Let us improve regulation and its functioning, but let us also consider improving people’s culture and education. This latter may be more expensive, but it is also more enduring” (Melé,, 2009:64).

#### Current Ethical Banking

Recent years have seen a proliferation of new forms of financial business that seek to make economic profitability compatible with respect for human rights and the environment. One such business is ethical banking, which invests only in projects offering value added to society, mainly from an educational, cultural, environmental, and/or social perspective. In this context, ethics is understood as a science aimed at steering human action in a rational direction (Cortina, 1994:17). Many papers have looked at ethical banking and demonstrated the important role it plays as an independent, differentiated financing activity (Lynch, 1991; Cowton and Thompson, 2001; Alsina, 2002; Fergeson, 2004; Barbu and Vintila, 2007; Buttle, 2008; Baranes, 2009; Cowton, 2010).

In the economic and financial arena, ethics must fulfill the important mission of ensuring equal access by agents to all types of goods and services. To achieve this, the financial system must promote the achievement of monetary and financial stability and effectively contribute to it, in accordance with its purpose of optimizing the use of financial resources, by channeling the savings generated by surplus spending units to borrowers or deficit spending units (Calvo and Martín de Vidales, 2014; Calvo et al., 2014:1). In short, it must facilitate real or productive economic activity and foster overall rather than just individual well-being, which is the first principle of financial ethics (Camacho Laraña, 1996:35).

To date, the concern for ethics in the financial system has led to two main developments: the establishment of ethical banks and the launch of ethical or socially responsible investment (SRI) funds, that is, funds in which certain social values (usually related to environmental quality and quality of life) are given precedence over strictly financial ones (risk-return). The ethical banking model could be included in the market for SRI funds. Ethical banks manage their customers’ money, allocating it to investments and projects based on environmental, social, and governance (ESG) criteria with the aim of creating something of social utility for the surrounding community, above and beyond the mere pursuit of profit. This social purpose refers to the social return on the invested capital and to the social responsibility of the investor (Spansif, 2013:6; Benito Hernández, 2014).

Europe has had several experiences with ethical banks, which have positioned themselves at the epicenter of some of the Old Continent’s largest economies, especially in innovative sectors with high growth potential. Some have joined the Global Alliance for Banking on Values (GABV), established in 2009 with a view to examining the financial dynamics of capital and the systems for measuring the impact on the overall, environmental, and financial development of sustainable banks. In addition, the GABV established, for the first time, a precise definition of sustainable banking, endorsing the following principles (Global Alliance for Banking on Values [GABV], 2012):

- A triple bottom line (social, environmental, and economic) approach at the heart of the business model. To achieve a social return, ethical banks must finance economic activities that offer social value added and eschew investments in speculative projects or companies meeting negative criteria. Achieving an economic return (i.e., making a profit) requires good bank management. Since ethical banks do not usually distribute profits to shareholders, and, when they do, they do so only in very limited amounts, any profit should be residual (Green, 1989; Lynch, 1991; Cowton and Thompson, 2001; Alsina, 2002; Fergeson, 2004).
- Grounded in communities, serving the real economy, and enabling new business models to meet the needs of both.
- Long-term relationships with clients and a direct understanding of their sectors of economic activity and the risks involved.
- Long-term goals, self-sustaining, and resilient to outside disruptions.
- Transparent and inclusive governance.
- All these principles should be embedded in the bank’s culture.

Recognition of the institution as a bank or credit institution by the national authorities is essential (Iturrioz et al., 2005; Valor, 2005; Valor et al., 2007). This dimension is important to distinguish between ethical banks and other financial experiences, such as solidarity programs or foundations that depend on banks but do not operate as real financial institutions. Some traditional banks have foundations that might in and of themselves meet ethical criteria, but are not, strictly speaking, credit institutions, since they depend on the bank’s business, which probably has a different kind of social impact. Ethical commitments must thus affect all aspects of the bank and not just part of it or its activities (San-Jose et al., 2011:152–153).

### Why Do Consumers Choose Ethical Banking? An Analysis of the Spanish Case

In Spain, investing according to ethical, sustainability, and/or social responsibility criteria remains considerably less developed than in many northern European countries, despite having become, in recent years, one of the levers for advancing toward a more sustainable economic model able to overcome the effects of the crisis. Indeed, this form of investment remains a niche type of investment dominated by a small number of large institutional investors, which together account for 97% of the assets managed according to ESG criteria (Novaster, 2015:3). Of these, the most active participants are employment pension plans, which are the most important drivers of SRI in Spain, as SRI retail funds have only a very marginal presence, due both to the sparse selection currently offered by the main financial institutions and a lack of knowledge on the part of private investors.

Accordingly, no ethical banks have yet been founded in the Spanish financial system. Therefore, the influence of this type of ethical action can be said to be limited to just three fronts. The first is the launch by some traditional banks of financing lines based on the granting of microcredits, mainly targeted at entrepreneurs, personal and family development projects, microenterprises seeking to meet social needs, and ecologically sustainable or environmentally friendly projects. CaixaBank, BBVA, and the ICO have been quite active in this business segment for many years. The second is the opening in Spain of branches of different ethical banks, specifically, Triodos Bank and Fiare Banca Etica. Finally, the third is the marketing by organizations that are not financial institutions – mainly, service cooperatives – of savings products and ethical financing. In this sphere, the initiatives undertaken by Oikocredit and Coop57 stand out. The most important aspects of the latter four institutions are explained below.

(1) *Triodos Bank.* Triodos Bank was established in the Netherlands in 1980, following the creation of the Triodos Foundation in 1971, as a way to channel donations and loans to companies excluded from the financial sector. Since then, Triodos has grown and expanded its original approach. Through its extensive European branch network (it currently also has operations in Belgium, the UK, Spain, and Germany), it finances companies, institutions, and projects that add clear social, environmental, and cultural value with the support of depositors and investors who wish to encourage socially responsible businesses and organizations and a sustainable society. To this end, its business model seeks to strike a balance between people’s quality of life, care of the planet, and economic profit, what is known as the triple bottom line approach, or the ‘three Ps’ (People, Planet, Profitability) (Triodos Bank, 2008–2015).
(2) *Fiare Banca Ética.* Fiare Banca Ética is the result of the merger of two ethical finance projects: Banca Popolare Etica, a cooperative bank founded in the city of Padua (Italy) in 1999 from volunteer associations and cooperatives from northeastern Italy, and the Fiare Foundation, established in 2003 in the Basque Country to build a movement of active citizenry with a view to laying the foundations for ethical and cooperative banking in Spain. In 2005, the two institutions signed an agent agreement, and financial intermediation began in Spain on October 31 that year. In short, this institution was founded to serve as a tool to promote social transformation, as its members’ savings deposits allow it to finance projects linked to the social welfare system (especially health and social services, social housing, and social microcredits), energy efficiency, the environment, local agriculture, international cooperation, social and cultural facilitation, and fair trade.
(3) *Oikocredit.* According to Ban Ki-Moon, Secretary-General of the United Nations, service cooperatives “are a reminder to the international community that it is possible to pursue both economic viability and social responsibility” (Oikocredit, 2014–2015a,b). In this business segment, the international financial cooperative Oikocredit, originally called the Ecumenical Development Cooperation Society (EDCS), plays a prominent role. It was founded in 1975 by the World Council of Churches with the aim of offering religious institutions an alternative investment instrument targeted at disadvantaged people. Despite these origins, Oikocredit’s investment criteria were never based on religious principles. Today, with the same goal of achieving inclusive finance, it has a decentralized structure that reaches from its headquarters in Amersfoort (Netherlands) to much of the planet, financing 809 organizations in 69 countries. In Spain, it operates through three support associations in the Basque Country (since 1997), Catalonia (since 2000), and Seville (since 2007), providing loans and investment to microfinance institutions and fair trade organizations, agricultural cooperatives, and small to medium-sized enterprises with a social justice and environmental dimension, that is, organizations that contribute to sustainably improving the quality of life of low-income earners and communities.
(4) *Coop 57.* Finally, attention should be called to the initiative carried out in this sphere by the ethical and charitable financial services cooperative Coop57, whose activity began in Catalonia with workers’ struggle to keep their jobs at the Editorial Bruguera publishing house. A group of these workers used part of the compensation they had received in the lay-off to create a fund to promote economic projects aimed at creating quality jobs, especially through the application of cooperative models. With this fund, Coop57 was founded, on June 19, 1995. Although at first it was closely linked to associationist cooperativism, it gradually expanded its social base to include other types of social and solidarity economy organizations. Indeed, in 2005, Coop57 adopted a networked growth strategy throughout Spain based on the interest that it had stirred in other regions of the country, which used the business model and legal and technical structure of the parent company. Currently, in addition to its headquarters in Barcelona, it has regional sections with their own management and participation structures in Aragon (since 2005), Madrid (since 2006), Andalusia (since 2008), and Galicia (since 2009). Its main goal is to collect people’s savings in order to channel them toward the financing of social and solidarity economy organizations that promote stable quality employment, foster cooperativism, associationism, and solidarity in general, and promote sustainability and food and energy sovereignty, based on ethical and charitable principles. In short, although Coop57 carries out a financial activity, it does so not in pursuit of an economic goal, but rather a social one: the financing of projects that add value to their communities and society as a whole.

Although the presence of ethical banking operations in Spain remains fairly small, numerous experts have argued that the silent revolution of ethical, social, and charitable finance has begun (Sanchis, 2016) and that it is having a positive impact on growth.

## Results

First of all, it is important to consider that the lack of homogeneous data sources for this sector, statistical gaps, and the absence of a uniform nomenclature for both the activities themselves and the purpose thereof at the analyzed institutions (Coop57, 2007–2015; Fiare Banca Ética, 2008–2015; Triodos Bank, 2008–2015; Oikocredit, 2014–2015a,b) require an initial approximation of the sector in order to measure the impact of this banking model on the Spanish economic system (Supplementary Data Sheet 1). For the empirical research, the whole econometric models were technically validated, and all structural and random hypotheses were supported.

The first aim of the research was to measure the development of ethical banking in the Spanish financial sector compared to traditional banking. To this end, all ethical banking in Spain was represented through the analysis of two variables, which were selected on the basis of relevance and ease of comparison with traditional banking: deposits and loans. In order to ensure conceptual consistency and enable the comparison of ethical banking and traditional banking, the heading “deposits” includes contributions that can be used to fund grants and projects (from own funds to deposits and lending investments), while the heading “loans” is the sum of allocated amounts (credits, loans, social projects, etc.).

**FIGURE 1:**
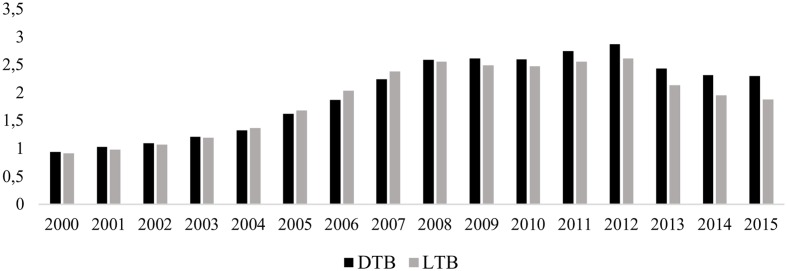
**Deposits (DTB) and loans (LTB) at traditional banks (€ billion).** DTB, deposits at traditional banks; LTB, loans at traditional banks.

**FIGURE 2:**
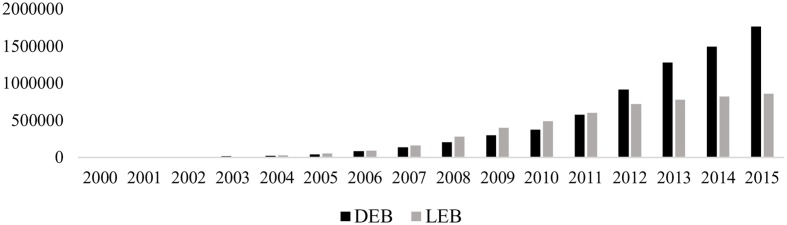
**Deposits (DEB) and loans (LEB) at ethical banks (€ thousand).** DEB, deposits at ethical banks; LEB, loans at ethical banks.

**Figure 1** shows how, following the onset of the financial crisis in 2007 and the subsequent reform of the Spanish banking system, loans declined faster than managed deposits in the traditional banking segment. In contrast, in the ethical banking segment, growth in loans outstripped deposit growth until 2012 (see **Figure 2**). Since then, the trend has reversed, with loans showing only modest linear growth in contrast with soaring deposits.

**FIGURE 3:**
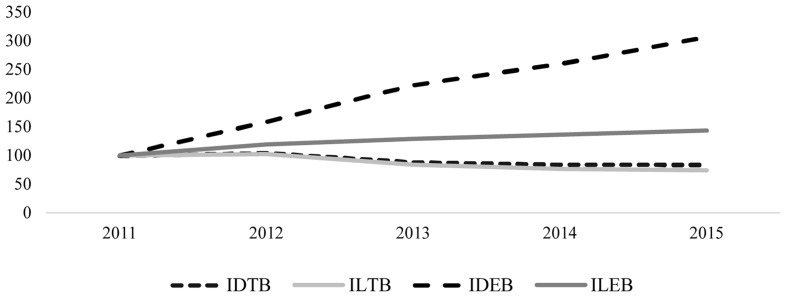
**Deposits and loans at TBs and EBs (Index: 2011 = 100)**.

**Figure 3** shows the 2011–2015 series. As in prior years these indices would yield very high rates, making it impossible to compare the two ratios, so in order to solve this problem, an index number were calculated. A comparative study of deposits and loans shows that, in traditional banking, the two headings have behaved roughly the same. In contrast, ethical banking has grown faster, with growth in customer deposits being particularly strong.

**FIGURE 4:**
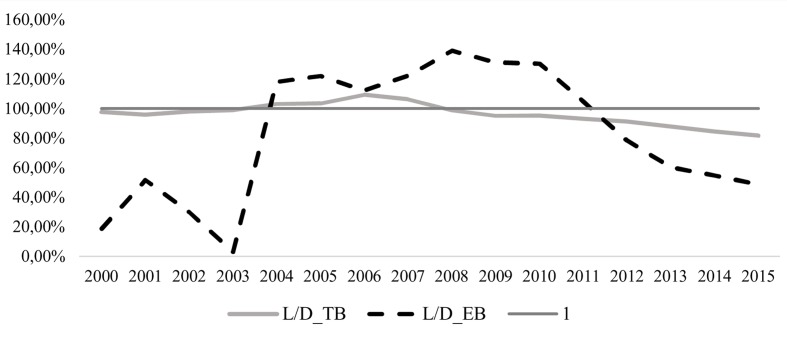
**(Loans/Deposits) in TB and EB**.

Finally, a comparative ratio of loans and deposits for both types of banks, reflecting both of the preceding points, since 2007 shows that the banking system in general has become more conservative (see **Figure 4**). In traditional banking, not all existing available funds are allocated to loans. The same has been true in ethical banking since 2012, but in a much more pronounced way.

Both for the comparative analysis and to illustrate the change in behavior that has led consumers to trust ethical banks, several simple but fairly representative econometric models were considered.

First, a model was proposed to analyze the recent evolution of ethical banking, taking consumer behavior in the sector into account (**Table 1**).

**Table 1:** Econometric model explaining the evolution of loans granted by ethical banks.

Dependent variable: LEB				
Method: Least squares				
Date: 09/19/16 Time: 11:55				
Sample: 2000 2015				
Included observations: 16				

**Variable**	**Coefficient**	**Std. Error**	***t*-Statistic**	**Prob.**

*C*	430391.7	65991.29	6.521948	0.0000
DEB	0.232091	0.039341	5.899504	0.0001
EUR	-75139.18	17144.48	-4.382704	0.0011
SI	-115.0631	60.92062	-1.888739	0.0856
F06	182388.5	44277.37	4.119225	0.0017

*R*-squared	0.988120	Mean dependent var.		330842.6
Adjusted *R*-squared	0.983800	SD dependent var.		333402.6
SE of regression	42435.87	Akaike info criterion		24.39968
Sum squared resid.	1.98E+10	Schwarz criterion		24.64112
Log likelihood	-190.1975	Hannan–Quinn criter.		24.41205
*F*-statistic	228.7244	Durbin–Watson stat.		1.607949
Prob(*F*-statistic)	0.000000			


In other words, the economic causes that lead consumers-investors to increase the volume of ethical banking were proposed empirically.

In addition to transparency and the social reasons discussed above, certain economic and financial factors have significantly helped to drive ethical banking business in recent years and could serve as an initial hypothesis.

(1) In the Spanish economy, depositors (investors) primarily seek returns in:
  (a) securities in primary and secondary markets (stock exchange),
  (b) interest on deposits in traditional banking and other financial investments (funds), and,
  (c) the growing productive economic sector.
(2) In Spain, until 2007, the construction sector was a refuge for investors, offering very high returns. However, that year, real estate activity collapsed. The EURIBOR, as a reference rate, likewise began to decline beginning in 2007, making it less attractive to investors.
(3) The stock index is also not a particularly obvious choice for investors. It began to fall in 2008, although with ups and downs. It rose somewhat in 2009, and then fell again from 2010 to 2013, when it rallied slightly, before declining again in 2014, reaching 2004 levels in 2015.
(4) It was precisely in 2007 that ethical banking began its upward trend. Possibly, many depositors with financing capacity may have chosen to seek a return in this sector given the social characteristics and transparency of this type of banking.

**FIGURE 5:**
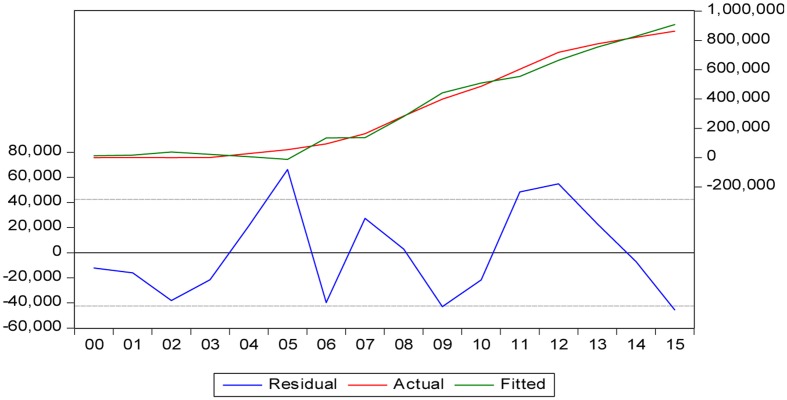
**Fit of the explanatory model of loans granted by ethical banks**.

In light of the above, the paradigm was obtained with the following econometric model (see **Table 1** and **Figure 5**), where:

LEB: Loans granted by ethical banks (€ thousand, current prices).

DEB: Deposits at ethical banks (€ thousand, current prices).

EUR: EURIBOR.

SI: Stock index.

F06: Dummy variable that corrects a technical problem in the structural change in the model.

Specification⁢1: LEB^=β^0+(β^1*DEB)+(β^2*EUR)+(β^3*SI)+(β^4*F06)

Estimate⁢1: LEB^=430391.7+(0.232091*DEB)−(75139.18^*EUR)−(115.0631*SI)+(182388.5*F06)

In conclusion, ethical banking provides financing based on the level of deposits (direct relationship). At the same time, the model (with a fit of 98.38%) shows that the situation of declining interest rates and a falling stock index justified the increase in these loans, subject to deposit levels.

**FIGURE 6:**
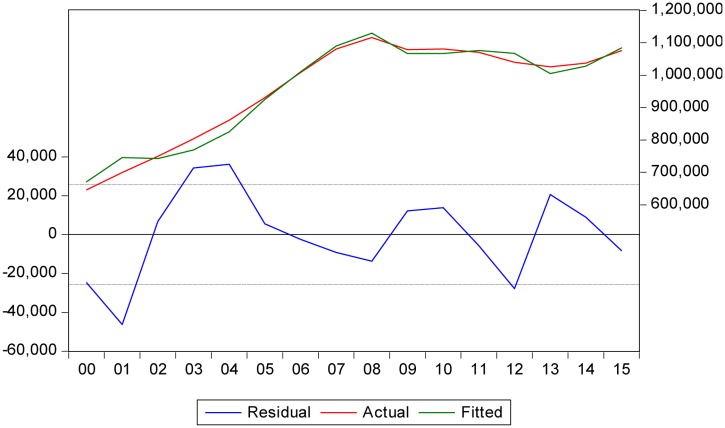
**Fit of inputs (capital and labor) with the evolution of the Spanish GDP**.

The next question is whether this consumer choice is favorable or unfavorable to the country’s economic system. The following model of the banking sector is revealing in this regard. It shows the importance of ethical banking and traditional banking to GDP, including both the capital input (through deposits at traditional and ethical banks) and the labor input (from the complementary perspective of the unemployment rate or UR). Considering the justification of the proposed specification, as a function of aggregate production (GDP), and the inputs of capital (LEB, LTB) and labor (UR), yields the following econometric model (see **Table 2** and **Figure 6**):

**Table 2:** Econometric model of inputs (capital and labor) in the Spanish GDP.

Dependent variable: GDP				
Method: Least squares				
Date: 09/22/16 Time: 13:21				
Sample: 2000 2015				
Included observations: 16				

**Variable**	**Coefficient**	**Std. Error**	***t*-Statistic**	**Prob.**

*C*	776319.6	51021.52	15.21553	0.0000
LEB	0.360935	0.057430	6.284781	0.0001
LTB	175.4056	18.29093	9.589756	0.0000
UR	-19138.02	3162.886	-6.050809	0.0001
F08	65824.71	39587.12	1.662781	0.1246
*R*-squared	0.979597	Mean dependent var.		956495.5
Adjusted *R*-squared	0.972178	SD dependent var.		154450.4
SE of regression	25762.37	Akaike info criterion		23.40152
Sum squared resid.	7.30E+09	Schwarz criterion		23.64296
Log likelihood	-182.2122	Hannan–Quinn criter.		23.41389
*F*-statistic	132.0339	Durbin–Watson stat.		1.289204
Prob(*F*-statistic)	0.000000			


Specification2:GDP^=γ^0+(γ^1*LEB^)+(γ^2*LTB)+(γ^3*UR)+(γ^4*F08)

Estimate2:GDP^=776319.6+(0.360935*LEB)+(175.4056*LTB)−(19138.02*UR)+(65824.71*F08)

Where:

GDP: Gross Domestic Product (€ million, current prices).
LEB: Loans granted by ethical banks (€ thousand, current prices).
LTB: Loans granted by traditional banks (€ billion, current prices).
UR: Unemployment rate (%).
F08: Dummy variable correcting for technical problems in the model’s structural change.

The model conforms to economic theory and the signs are consistent with macroeconomic performance. The levels of loans granted by both ethical and traditional banks (capital input) positively impacted the growth of the Spanish economy. As expected, the unemployment rate (labor input) negatively impacted growth, i.e., the higher the unemployment rate, the lower the economic growth.

## Discussion

Ethical consumption is a growing movement, and consumers are increasingly committed to ethical factors when forming opinions about products and making purchase decisions (Shaw and Shui, 2002; De Pelsmacker et al., 2005). However, several studies have revealed the existence of a possible gap between purchase intentions and actual purchasing behavior (ethical purchasing gap), showing that ethically conscientious consumers rarely shop ethically (Bray et al., 2011; Goyal and Joshi, 2011; Papaoikonomou et al., 2011; Carrington et al., 2014).

The ethical issues involved in banking practices have become an important aspect that the nations of the world must take into consideration, especially in light of the incessant bank failures and ensuing loss of customers’ deposits (Asikhia, 2016). However, the ethical banking sector has grown, not only as a result of the bad banking and financial practices of recent years, but also because of the confluence of the economic crisis, banking reform, declining returns for investors through traditional channels, and, especially, the ethical and social commitment that has recently begun to emerge in many activities.

Based on the preceding empirical analysis, we can offer an initial response to the questions posed at the start of this paper.

1. Can bank consumers-investors change the characterization of the banking system?
  By standardizing the coefficients^1^ of the first estimated model (**Table 1**), we can see the unit effect of each explanatory variable (deposits at ethical banks, Euribor, and stock index) on the endogenous variable (loans granted by ethical banks), giving:
  Although deposits at ethical banks (β^EBD*=0.41055) explain the behavior of ethical banking loans to a greater degree than either the Euribor (β^EUR*=0.4016) or the stock index does alone, they do so in a smaller proportion and with the inverse sign compared to the Euribor and stock index taken together (β^SI*=0.09807). Therefore, when both the Euribor and the stock index increase, deposits at ethical banks will decrease due to the greater profitability of deposits at traditional banks, resulting in a decline in ethical banking loans at high values.
  In short, it can be concluded that, if the Euribor continues to fall as it has to date, banking consumers-investors could indeed change the characterization of the banking system by choosing the investments offered by ethical banks through deposits.

2. Can ethical banking gain ground on traditional banking? Is it really effective?
  Interpreting the results of the second estimated model (**Table 2**) to determine the extent to which each variable (loans granted by ethical banks, loans granted by traditional banks, and the unemployment rate) contributes to the evolution of GDP, and again standardizing the coefficients, yields:
  In this case, the unemployment rate (β^UR*=0.794931) explains GDP behavior to a greater extent than loans, whether granted by ethical or traditional banks (β^LEE*=0.7791, β^LTB*=0.69925).
  However, when the impact of loans is considered in isolation, loans granted by ethical banks explain GDP behavior to a greater degree, although the difference is not huge (11.42%). This means that an increase in ethical banking loans will be more effective for GDP growth than an increase in traditional banking loans, as long as the Euribor and stock index hold steady or decline (conclusion obtained and discussed from **Table 1**).
  In sum, if the Euribor continues to register low values, as it has to date, ethical banking could gain ground on traditional banking, as it is more effective at increasing GDP.

3. Are the principles of ethical banking fulfilled?
  **Figures 1**–**5** could cast doubt on whether ethical banking faithfully fulfills some of its basic principles in terms of its “social and environmental bottom lines” and operating “at the service of the real economy”.
  The foregoing analysis showed that deposits managed by ethical banks are growing at a far higher rate than the loans they grant. At first glance, this finding poses a twofold question regarding the sector’s future viability and whether it may not, in the long term, conform more closely to the traditional banking business model.
  However, the existing gap between deposits and loans may be justified if ethical banks are allocating the financing collected through deposits to corporate purposes that they share with traditional banks (e.g., hiring staff, the financing of new physical branches of institutions in this business segment, etc.). In that case, it would make realistic economic sense, as a sector that creates jobs and capital input, thereby generating more efficient and sustained economic growth. It must be remembered that the primary labor of this type of banking is the financing, through financial intermediation, of social and solidarity economy projects that promote, among other things, the creation of stable, quality employment, sustainable development, food and energy sovereignty, the social inclusion and employment of vulnerable groups, international cooperation, fair trade, culture, education, and civic engagement.
  Therefore, in addition to increasing the economic bottom line, as traditional banking does, by creating jobs and wealth through the acquisition or leasing of office space, it would continue to ensure the sustainability and social purposes that characterize its ideology, resulting in an improvement in people’s quality of life and better use of the available environmental resources.
  However, any conclusion regarding the consistency of ethical banking will need to await a time when both the Euribor and stock index are rising.

## Author Contributions

All authors listed, have made substantial, direct and intellectual contribution to the work, and approved it for publication.

## Conflict of Interest Statement

The authors declare that the research was conducted in the absence of any commercial or financial relationships that could be construed as a potential conflict of interest.

## Footnotes

1 β^*(Xj)=β^(Xj)•(SDXj/SDY); where - SD is the standard deviation of the variable. - X: explanatory or exogenous variable, and - Y: variable to be explained or endogenous variable.
