# Supplementary material for: Assessing the Growth of Ethical Banking: Some Evidence from Spanish Customers
Source: Front Psychol. 2017 May 24;8:782. doi: 10.3389/fpsyg.2017.00782 (PMC5443157; doi:10.3389/fpsyg.2017.00782)
Supplement: Supplementary file 1 [file Data_Sheet_1.docx]

**STATISTICAL APPENDIX**

|  | DTB (*10^9^)^1^ | LTB  (*10^9^)^1^ | DEB (*10^3^)^2^ | LEB  (*10^3^)^2^ | SI^1^ | GDP**^1^** | UR^1^ | EUR^3^ |
| --- | --- | --- | --- | --- | --- | --- | --- | --- |
| **2000** | 939 | 916 | 364 | 69 | 1016.32 | 646,250 | 13.9 | 4.01 |
| **2001** | 1,028 | 985 | 597 | 309 | 824.40 | 699,528 | 10.63 | 4.25 |
| **2002** | 1,096 | 1,074 | 768 | 231.09 | 633.99 | 749,288 | 11.61 | 4.25 |
| **2003** | 1,214 | 1,199 | 17082 | 550.09 | 807.98 | 803,472 | 11.37 | 4.25 |
| **2004** | 1,329 | 1,371 | 23360 | 27595 | 959.06 | 861,420 | 10.53 | 4.25 |
| **2005** | 1,629 | 1,686 | 44682 | 54632 | 1,156.21 | 930,566 | 8.71 | 4.25 |
| **2006** | 1,869 | 2,042 | 83780 | 94214 | 1,554.93 | 1,007,974 | 8.26 | 4.25 |
| **2007** | 2,244 | 2,387 | 133488 | 163388 | 1,637.03 | 1,080,807 | 8.57 | 4.25 |
| **2008** | 2,593 | 2,561 | 203694 | 283895 | 975.97 | 1,116,207 | 13.79 | 3.55 |
| **2009** | 2,619 | 2,496 | 303256 | 398928 | 1,241.72 | 1,079,034 | 18.66 | 1.31 |
| **2010** | 2,599 | 2,474 | 373437 | 487242 | 1,003.73 | 1,080,913 | 20.11 | 1 |
| **2011** | 2,750 | 2,555 | 574768 | 602798 | 857.65 | 1,070,413 | 22.56 | 1.24 |
| **2012** | 2,871 | 2,617 | 915628 | 719163 | 824.70 | 1,039,758 | 25.77 | 0.88 |
| **2013** | 2,433 | 2,138 | 1283263 | 776636 | 1,011.98 | 1,025,634 | 25.73 | 0.54 |
| **2014** | 2,318 | 1,956 | 1497917 | 821471 | 1,042.46 | 1,037,025 | 23.7 | 0.16 |
| **2015** | 2,306 | 1,884 | 1765497 | 862361 | 965.13 | 1,075,639 | 20.9 | 0.05 |

DTB: Deposits at traditional banks.

LTB: Loans granted by traditional banks.

DEB: Deposits at ethical banks.

LEB: Loans granted by ethical banks.

SI: Stock index (baseline: 1985).

GDP: Gross Domestic Product (€ million, current prices).

UR: Unemployment rate (% of active population).

EUR: EURIBOR interest rate (%)

Statistics sources:

1. Instituto Nacional de Estadística (INE). 2016. [National Statistics Institute]. Ine.es
2. Aggregate result of the data provided, for Spain, by: Fiare, Triodos, Coop57, and Oikocredit.
3. Banco de España (2000-2015).
